# Radiomics Nomogram Based on High-*b*-Value Diffusion-Weighted Imaging for Distinguishing the Grade of Bladder Cancer

**DOI:** 10.3390/life12101510

**Published:** 2022-09-28

**Authors:** Cui Feng, Ziling Zhou, Qiuhan Huang, Xiaoyan Meng, Zhen Li, Yanchun Wang

**Affiliations:** Departments of Radiology, Tongji Hospital, Tongji Medical College, Huazhong University of Science and Technology, Wuhan 430030, China

**Keywords:** bladder cancer, diffusion-weighted imaging, high *b*-value, radiomics nomogram, grading

## Abstract

Background: The aim was to evaluate the feasibility of radiomics features based on diffusion-weighted imaging (DWI) at high *b*-values for grading bladder cancer and to compare the possible advantages of high-*b*-value DWI over the standard *b*-value DWI. Methods: Seventy-four participants with bladder cancer were included in this study. DWI sequences using a 3 T MRI with *b*-values of 1000, 1700, and 3000 s/mm^2^ were acquired, and the corresponding ADC maps were generated, followed with feature extraction. Patients were randomly divided into training and testing cohorts with a ratio of 8:2. The radiomics features acquired from the ADC_1000_, ADC_1700_, and ADC_3000_ maps were compared between low- and high-grade bladder cancers by using the Wilcox analysis, and only the radiomics features with significant differences were selected. The least absolute shrinkage and selection operator method and a logistic regression were performed for the feature selection and establishing the radiomics model. A receiver operating characteristic (ROC) analysis was conducted to assess the diagnostic performance of the radiomics models. Results: In the training cohorts, the AUCs of the ADC_1000_, ADC_1700_, and ADC_3000_ model for discriminating between low- from high-grade bladder cancer were 0.901, 0.920, and 0.901, respectively. In the testing cohorts, the AUCs of ADC_1000_, ADC_1700_, and ADC_3000_ were 0.582, 0.745, and 0.745, respectively. Conclusions: The radiomics features extracted from the ADC_1700_ maps could improve the diagnostic accuracy over those extracted from the conventional ADC_1000_ maps.

## 1. Introduction

Bladder cancer (BC) ranks as the sixth most common cancer and the ninth leading cause of cancer-specific death in males worldwide [1]. In the prognosis and management strategy of BC, histologic grading has been recognized as a crucial factor to be considered [2]. BC is graded as low- or high-grade depending on the degree of nuclear anaplasia and architectural abnormalities [3]. Low-grade BC has demonstrated a lower rate of recurrence and stage progression compared to high-grade BC. The accurate assessment of the degree of BC tumor cell differentiation is essential not only for selecting the best treatment options, but also for sparing patients from unnecessary invasive treatment for low-risk non-muscle-invasive bladder cancer, reducing the likelihood of local recurrence and stage progression while maintaining the quality of life [4].

Transurethral resection of bladder tumor (TURBT) is considered a standard method to establish histologic grade [5,6]. However, inaccurate grading occurs in up to 15% of cases due to sampling errors and the heterogeneous characteristics of tumors [7,8]. Furthermore, due to the high cost and the need for repeat operations of TURBT [9], accurate and noninvasive techniques are needed to assess the aggressiveness of BC.

By using the diffusion of water molecules as a probe, diffusion-weighted imaging (DWI) can reveal tissue microstructural changes in vivo, particularly in cancer [10,11]. Among many quantitative parameters that DWI can produce, the apparent diffusion coefficient (ADC) has been investigated most extensively for characterizing cancerous tissues, including bladder cancer [12,13,14,15]. Its routine clinical use, however, has been hampered by the considerable overlap of ADC values among different tumor grades [16,17]. In clinical practice, ADC maps are typically calculated from DWI with *b* = 0 and 1000 s/mm^2^. The clinical application of high-*b*-value DWI is limited because of the inferior signal-to-noise ratio (SNR) in 1.5 T or lower-field-strength MR systems. Recently, the popularization of the 3.0 T MR systems in medical units has enabled for us to acquire high-*b*-value DWI (e.g., *b* = 3000 s/mm^2^) with an acceptable SNR within a clinically acceptable data acquisition time frame. Recently, many studies have indicated that ADC maps obtained from high-*b*-value DWI are more effective than those obtained from standard-*b*-value DWI in several aspects [18,19,20].

Radiomics can extract a large number of advanced quantitative features from medical images and it has been used in the evaluation of tumor staging [21,22,23], grading [24], and predicting the recurrence of bladder cancer [25]. To the best of our knowledge, little research has been done to establish the application of radiomics nomogram based on high-*b*-value DWI in the preoperative evaluation of the grade of BC. Hence, this study aimed to explore the potential feasibility of radiomics nomogram based on DWI at high *b*-values (*b* = 1700 and 3000 s/mm^2^) for grading bladder cancer and to compare the possible advantage of high-*b*-value DWI over the standard-*b*-value (*b* = 1000 s/mm^2^) DWI.

## 2. Materials and Methods

### 2.1. Patient Characteristics

**Figure 1:**
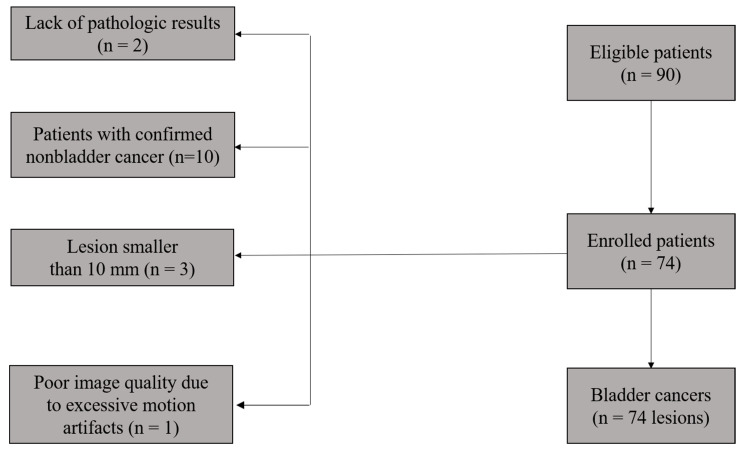
Flowchart of the study population.

This retrospective study was approved by the Institutional Review Board of our hospital, and informed written consent was waived. Ninety patients with suspected or confirmed bladder lesions (e.g., by ultrasonography or CT) between July 2014 and November 2019 were enrolled in this study. The inclusion criteria were (i) the availability of histopathological confirmation through TURBT or cystectomy and (ii) no treatment prior to the MR examination. The exclusion criteria consisted of (i) the unavailability of histopathological confirmation through TURBT or a cystectomy after the MRI examination (*n* = 2), (ii) confirmed nonbladder cancer (*n* = 10), (iii) poor image quality due to excessive motion artifacts (*n* = 1), or (iv) a diameter of tumor less than 1 cm (*n* = 3). With these criteria, a total of 74 patients (63 males, 11 females; median age, 61 ± 10 years; age range, 37–79 years) were finally included in this study. The flowchart of this study population is shown in Figure 1.

### 2.2. Image Acquisition

All participants underwent MR examinations on a 3 T scanner (Discovery MR750; GE Healthcare, Milwaukee, Brookfield, WI, USA) in the supine position with a 32-channel torso phased-array coil. The imaging protocol included axial fast spin-echo T1-weighted, axial fast recovery fast spin-echo T2-weighted, sagittal fast spin-echo T2-weighted, and diffusion-weighted imaging sequences. The acquisition parameters of each nondiffusion imaging sequence were as follows: (i) axial T1-weighted imaging: repetition time/echo time = 528/6.8 ms, field of view (FOV) = 340 × 340 mm^2^, matrix size = 320 × 256, and echo train length = 4; (ii) axial fast recovery T2-weighted imaging: repetition time/echo time = 3780/75 ms, FOV = 340 × 340 mm^2^, matrix size = 320 × 256, and echo train length = 16; (iii) sagittal T2-weighted imaging: repetition time/echo time = 5500/75 ms, FOV = 240 × 240 mm^2^, matrix size = 320 × 320, and echo train length = 24. In all sequences above, a section thickness of 4 mm with an intersection gap of 1 mm was used together with 2 averages. A series of axial diffusion-weighted images were obtained using a single-shot spin-echo echo-planar imaging sequence with 4 *b*-values, respectively: 0_1_, 1000_4,_ 1700_6_, and 3000_8_ s/mm^2^, where the subscript denotes the number of averages. A Stejskal–Tanner diffusion gradient was applied along the three orthogonal directions in order to acquire trace-weighted images to eliminate the effects of diffusion anisotropy. The acquisition parameters for the DWI sequence were: repetition time/echo time = 2500/84 ms, FOV = 400 × 400 mm^2^, matrix size = 128 × 160, section thickness = 4 mm, and section gap = 1 mm.

### 2.3. Image Segmentation, Preprocessing, and Feature Extraction

For the patients with multifocal lesions, only the lesion with the largest diameter was accessed in this study. ITK-SNAP software (open source, www.itk-snap.org) was used for the manual segmentation. The volume of interest (VOI) covering the whole lesion was placed by delineating along the tumor border layer by layer on the DWI images (*b* = 1000 s/mm^2^) by one radiologist (Yanchun.Wang., with 6 years of experience in MRI diagnosis), and confirmed by another radiologist (Cui Feng, with 11 years of experience in MRI diagnosis).

The ADC was calculated by employing the monoexponential model. The signal attenuation *S* was produced with the following equation:

(1) $$S=S_{0}\exp\left(-bD\right)$$

where *S* is the signal intensity at a given *b*-value and *S*_0_ is the signal intensity without diffusion weighting, *b* is known as the *b*-value, and *D* is the diffusion coefficient. The maps of ADC_1000_, ADC_1700_, and ADC_3000_ were obtained by employing equation (1) using diffusion-weighted images with two different *b*-values (*b* = 0 and 1000 s/mm^2^, *b* = 0 and 1700 s/mm^2^, *b* = 0 and 3000 s/mm^2^, respectively).

After the tumor segmentation, the texture analysis was conducted by using the LIFEx package [26] (version 6.00; Inserm, Orsay, France; https://www.lifexsoft.org (accessed on 13 May 2020)). Forty-nine texture features extracted from each VOI were as follows: seven statistical indexes (mean, minimum, maximum, the 25th percentile, the 50th percentile, the 75th percentile, and standard deviation of gray levels); six first-order histogram features (skewness, kurtosis, excess kurtosis, entropy_log10, entropy_log2, and energy); thirty-two higher-order features including the gray-level co-occurrence matrix (GLCM), the neighborhood gray-level different matrix (NGLDM), the gray-level run-length matrix (GLRLM), and the gray-level zone-length matrix (GLZLM); and four shape indexes (sphericity, compacity, volume_mL, and volume_voxels) were extracted in this study. A detailed description of each texture feature is available in the technical appendix of LIFEx software [26].

Patients were randomly divided into training (80% of the total patients) and testing cohorts (20% of the total patients) using the “sample” function (with seed set as 120) in R software.

### 2.4. Feature Selection and Model Building

The feature selection was performed in the training cohort using the following 2-step procedures: (1) features with obvious significance in differentiating low- and high-grade bladder cancer were selected by performing a Wilcoxon analysis in which a significant level of *p* < 0.1 was set [27] and (2) the least absolute shrinkage and selection operator (LASSO) method [28] was further applied to reduce the dimensionality of the features. An optimal LASSO penalty was acquired by minimizing the mean square error with a 10-fold cross-validation.

**Figure 2:**
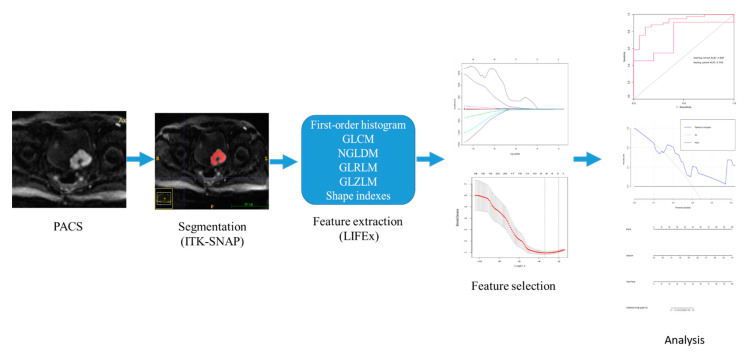
The radiomics workflow in this study.

The best subset of features was then used to develop the radiomics models. Models for discriminating low- from high-grade bladder cancer were built by using a logistic regression. The performance of the built model was assessed using the receiver operating characteristic curve in the testing cohort. The radiomics workflow in this study is shown in Figure 2.

### 2.5. Statistical Analysis

The dimensionality reduction and model building processes of the radiomics features, including the intensity histogram, GLCM, and GLRLM of each model, were implemented in R (Version 3.6.0, https://www.r-project.org/ (accessed on 21 April 2021)).

The Wilcoxon analysis, LASSO regression, and ROC curve analyses were performed by means of the “caret” and “pROC” packages, respectively. In all tests of differences, a *p*-value less than 0.05 was considered statistically significant.

## 3. Results

### 3.1. Clinical Characteristics

The patients’ clinical characteristics are summarized in Table 1. Among the 74 patients, 27 underwent radical cystectomy, 4 partial cystectomy, and 43 TURBT. The pathological T stage was determined according to the 2017 TNM system [29], yielding 41, 20, 3, and 10 stages T1, T2, T3, and T4 patients, respectively. The tumors were classified as low-grade in 22 patients and high-grade in 52 patients according to the 2016 World Health Organization classification system [30]. The training cohort consisted of 58 patients (high grade, 41; low grade, 17) and the testing cohort consisted of 16 patients (high grade, 11; low grade, 5).

### 3.2. Feature Selection

**Figure 3:**
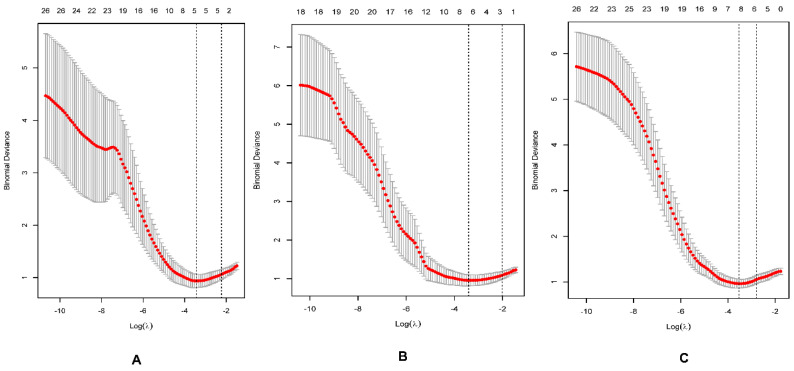
The best subset extracted from the least absolute shrinkage and selection operator (LASSO) regression method model consisting of 5, 7, and 7 features, corresponding to *b*−values of 1000 (**A**), 1700 (**B**), and 3000 (**C**) s/mm^2^, respectively.

In the training cohort, there were significant differences in 31, 26, and 44 features between the low and high grades of bladder cancer extracted from ADC_1000_, ADC_1700_, and ADC_3000_ maps, respectively. The best subset extracted from ADC_1000_, ADC_1700_, and ADC_3000_ maps by using the LASSO model consisted of five, seven, and seven features, respectively (Figure 3A–C and Table 2). The specific selected features included CONVENTIONAL_#std, CONVENTIONAL_#Q2, SHAPE_Sphericity (only for 3D ROI (nz > 1), GLRLM_RLNU, and NGLDM_Busyness for ADC_1000_; CONVENTIONAL_#std, SHAPE_Sphericity (only for 3D ROI (nz > 1), SHAPE_Compacity only for 3D ROI (nz > 1), GLRLM_HGRE, NGLDM_Busyness, GLZLM_HGZE and GLZLM_GLNU for ADC_1700_; HISTO_Skewness, SHAPE_Sphericity (only for 3D ROI (nz > 1), SHAPE_Compacity only for 3D ROI (nz > 1), GLCM_Correlation, GLRLM_LGRE, GLRLM_SRLGE, NGLDM_Contrast for ADC_3000_, respectively.

### 3.3. Performance of the Model

**Figure 4:**
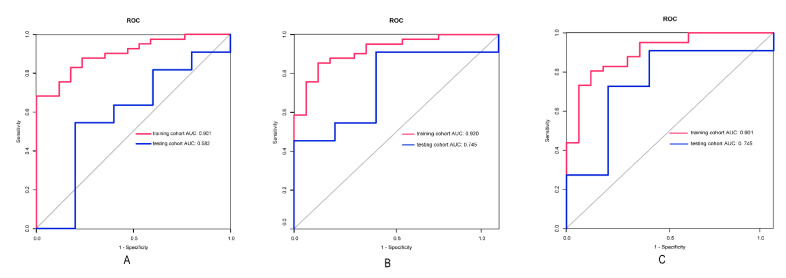
The ROC curve for the ADC1000 (**A**), ADC1700 (**B**) and ADC3000 (**C**) radiomics models for the training and testing cohorts.

The three radiomics models achieved good performance in the training and testing cohorts. The AUCs of the ADC_1000_ model, ADC_1700_ model, and ADC_3000_ model were 0.901 (95% confidence interval (CI): 0.825–0.977), 0.920 (95%CI: 0.849–0.990), and 0.901 (95%CI: 0.817–0.985) in the training cohorts. The AUCs of the ADC_1000_ model, ADC_1700_ model, and ADC_3000_ model were 0.582 (95% CI: 0.226–0.937), 0.745 (95%CI: 0.475–1.000), and 0.745 (95%CI: 0.451–1.000) in the testing cohorts. The detailed results are shown in Table 3. The ROC curves of the three models in the training and test models are shown in Figure 4.

## 4. Discussion

An accurate assessment of grade in BC is essential for urologists to develop appropriate strategies. Our results indicated that the radiomics features extracted from the ADC_1700_ maps could improve the diagnostic accuracy over that extracted from the conventional ADC_1000_ maps. We demonstrated the feasibility and possible superiority of using high-*b*-value DWI to assess the grade of bladder cancer.

Previous studies have reported the usefulness of texture analysis (TA) based on DWI to assess the grade of BC [24,31]. Razik et al. [31] indicated that a TA of DWI had excellent class separation capacity in differentiating high- from low-grade bladder cancer, which was consistent with our findings. However, the results of their article had an AUC maximum of 0.897 (*b*-value of 1500 s/mm^2^), which was higher than our AUC for a *b*-value of 1000 s/mm^2^, but lower than the AUC for a *b*-value of 1700 s/mm^2^, which also illustrated the advantages of high-*b*-values. Zhang et al. [24] indicated their maximum AUC value was 0.861 at a *b*-value of 1000 s/mm^2^, which was slightly lower than that of our study (AUC, 0.901). A possible reason for the discrepancy may be that more texture analysis parameters were extracted in our study, which was supposed to more accurately reflect the heterogeneity of bladder cancer in terms of the overall, local, and regional aspects [25]. It may also be because texture parameters were all extracted from the ADC maps in our study, while in the study of Zhang et al. [24], the texture parameters were extracted from the DWI and ADC maps.

Our result indicated that the radiomics parameters of ADC maps generated from high-*b*-value DWI (*b* = 1700 s/mm^2^ and 3000 s/mm^2^) provided a superior diagnostic performance compared with the standard *b*-value (*b* = 1000 s/mm^2^). Many previous studies have reported that DWI with a high *b*-value could enhance the clinical value in tumor grading and other aspects [18,19,20,32]. Kang et al. [18] indicated that the fifth percentile of the cumulative ADC histogram obtained at a high *b*-value (*b* = 3000 s/mm^2^) was the most promising parameter for differentiating high- from low-grade gliomas. Kwak et al. [18] indicated that the texture features extracted from high-*b*-value DWI images (*b* = 2000 s/mm^2^) produced better performance in the automated benign and malignant diagnosis of prostate lesions. These conclusions were similar to our findings. Intratumoral heterogeneity was an important consideration in tumor grading and predicting biological aggressiveness. Previous imaging studies on intratumoral heterogeneity were limited by the achievable voxel size. The advent of high-*b*-value DWI demonstrated a great potential for breaking this barrier and peeking into the voxels and was sensitive to tissue microstructures [11]. Therefore, ADC_1700_ and ADC _3000_ were supposed to reflect the heterogeneity of the tumor grade better. However, the TA of ADC _3000_ maps provided a less inferior diagnostic performance compared to that of ADC_1700_. The reason may be that the signal-to-noise (SNR) could be substantially reduced due to the increased diffusion-induced signal loss as well as the T2-induced signal attenuation caused by a longer TE to support the higher *b*-values.

It has to be declared that the diffusion-attenuated signal is described by the Stejskal–Tanner equation, assuming that the gradients are uniform. However, it has been indicated that the gradients are not uniform in almost any clinical or research MRI scanners, and the generalized Stejskal–Tanner equation has also been derived for nonuniform diffusion gradients [33]. It has been proven that the Stejskal–Tanner equation is still valid if the b-matrix can be calculated for each voxel separately, which is supposed to be achieved by using the b-matrix spatial distribution in DTI (BSD-DTI) technique [34,35].

This study has some limitations. First, the number of participants was moderate, and the distribution of pathologic grades was uneven with more high-grade than low-grade tumors. This may bias the statistical analysis and a further study of a larger population is required. Second, as a single-center retrospective study, the selective bias could not be avoided. Only one lesion was analyzed for the patients with multiple tumors, which could also cause a selection bias. Third, the small lesions (the diameter less than 1 cm) were not included due to the difficulty of drawing VOI, which led to the tumors evaluated in our study being relatively large. Fifth, systematic errors caused by the inhomogeneity of the gradients which affect the b-matrix, the eddy current effects, or other background interference do exit in the DWI measurements. However, the systematic errors are impossible to completely eliminate.

## 5. Conclusions

Our results indicated that the radiomics features extracted from ADC_1700_ maps could improve the diagnostic accuracy over those extracted from the conventional ADC_1000_ maps. We demonstrated the feasibility and possible superiority of using high-*b*-value DWI to assess the grade of bladder cancer, providing additional information for individualized treatment planning.

## Figures and Tables

**Table 1:** Clinical characteristics.

Variables	Characteristics
Age (years) *	61 ± 10 (37–79)
Gender	
Male	63 (85)
Female	11 (15)
No. of lesions	
Unifocal	56 (76)
Multifocal	18 (24)
Primary or recurrent tumors	
Primary	72 (97)
Recurrent	2 (3)
Tumor size (cm) *	3.1 ± 1.6 (1.0–10.1)
Pathologic stage	
T1	41 (55)
T2	20 (27)
T3	3 (4)
T4	10 (16)
Histologic grade	
Low	22 (30)
High	52 (70)
Lymph node metastasis	
Yes	13 (18)
No	61 (82)
Treatment methods	
TURBT	43 (58)
Radical cystectomy	27 (36)
Partial cystectomy	4 (5)

Note: Numbers in parentheses are percentages except where otherwise indicated. TURBT, transurethral resection of bladder tumor. * Numbers are means ± standard deviations, with ranges in parentheses.

**Table 2:** Calculation formula for radiomics signature.

Rad-Score	Variables	Coefficients
ADC1000	Intercept	1.199576464
	CONVENTIONAL_#std	−0.582534519
	CONVENTIONAL_#Q2	−0.615993908
	SHAPE_Sphericity (only for 3D ROI (nz > 1)	−0.77836837
	GLRLM_RLNU	0.340808516
	NGLDM_Busyness	0.029560922
ADC1700	Intercept	1.255336302
	CONVENTIONAL_#std	−0.54250182
	SHAPE_Sphericity (only for 3D ROI (nz > 1)	−1.110704238
	SHAPE_Compacity only for 3D ROI (nz > 1)	0.36442195
	GLRLM_HGRE	−0.538256985
	NGLDM_Busyness	0.050853414
	GLZLM_HGZE	−0.10091227
	GLZLM_GLNU	0.004710521
ADC3000	Intercept	1.234603905
	HISTO_Skewness	0.306892167
	SHAPE_Sphericity (only for 3D ROI (nz > 1)	−0.918088191
	SHAPE_Compacity only for 3D ROI (nz > 1)	0.869977739
	GLCM_Correlation	−0.62276962
	GLRLM_LGRE	0.139654102
	GLRLM_SRLGE	−0.098482715
	NGLDM_Contrast	−0.076828157

NOTE: GLRLM, the gray-level run-length matrix; NGLDM, the neighborhood gray-level different matrix; GLZLM, the gray-level zone-length matrix; GLCM, gray-level co-occurrence matrix.

**Table 3:** Diagnostic performances of the DWI of three *b*-values interpretation in differentiating high- from low-grade bladder cancer in the training cohort and the test cohort.

	AUC (95%CI)	Sensitivity	Specitivity
Training cohort (n = 58)			
ADC1000	0.901 (0.825–0.977)	0.683	1.000
ADC1700	0.920 (0.849–0.990)	0.854	0.882
ADC3000	0.901 (0.817–0.985)	0.805	0.882
Test cohort (n = 16)			
ADC1000	0.582 (0.226–0.937)	0.548	0.800
ADC1700	0.745 (0.475–1.000)	0.909	0.600
ADC3000	0.745 (0.451–1.000)	0.727	0.800

## Data Availability

The data presented in this study are available on request from the corresponding author. The data are not publicly available due to ethical concerns.
